# The mechanisms by which free heme exacerbates sepsis and the potential therapeutic targets

**DOI:** 10.3389/fimmu.2026.1743737

**Published:** 2026-07-15

**Authors:** Zheyang Sun, Junjie Cheng, Yuanjie Zhu, Lu Jiang, Min Li

**Affiliations:** 1 Department of Critical Care Medicine, The Fourth Affiliated Hospital of School of Medicine, and International School of Medicine, International Institutes of Medicine, Zhejiang University, Yiwu, China; 2 Department of Nephrology, Center for Regeneration and Aging Medicine, The Fourth Affiliated Hospital of School of Medicine, and International School of Medicine, International Institutes of Medicine, Zhejiang University, Yiwu, China; 3 Neuroscience Intensive Care Unit, The Second Affiliated Hospital, Zhejiang University School of Medicine, Hangzhou, Zhejiang, China

**Keywords:** cell death, disease tolerance, heme, PANoptosis, sepsis

## Abstract

Sepsis is marked by high morbidity and mortality rates, representing a significant contributor to the global disease burden. However, due to an incomplete understanding of its pathological mechanisms, current treatments remain predominantly symptomatic and supportive, lacking effective targeted therapies. Recent advances in research have brought the role of free heme in sepsis progression into focus. Free heme is now recognized as a critical mediator of sepsis exacerbation, with its biological properties and pathological mechanisms both playing pivotal roles. This review synthesizes evidence from foundational studies and clinical investigations to elucidate how free heme aggravates sepsis through direct cytotoxic effects and interactions with regulated cell death pathways. Furthermore, based on these mechanisms, potential therapeutic targets are proposed, alongside a summary of promising pharmaceutical candidates currently under investigation.

## Highlights

- Free heme is a critical factor exacerbating sepsis.
- The direct effects of free heme include cytotoxic actions, pro-inflammatory responses, and disruption of disease tolerance.
- Elucidating the relationship between free heme and regulated cell death.
- Potential therapeutic targets that may offer novel strategies for sepsis treatment.

## 1 Introduction

Globally, there were approximately 48.9 million cases of sepsis in 2017, with nearly 11.0 million sepsis-related deaths, accounting for 19.7% deaths that year (1). This proportion of sepsis-related deaths significantly exceeds previous estimates, suggesting that the disease burden of sepsis has been substantially underestimated (1). From 1990 to 2017, the incidence of sepsis seems to demonstrate a gradual downward trend (2), which may be attributed to improved early recognition and rapid intervention strategies. Nevertheless, sepsis continues to exhibit high incidence and mortality rates, indicating its persistent role as a major contributor to global health loss (2).

Sepsis-3 defines sepsis as “life-threatening organ dysfunction caused by a dysregulated host response to infection” (3). Thus, sepsis is not a singular disease but rather a syndrome arising from complex interactions between pathogens and hosts, emerging when an exaggerated systemic response to infection causes autologous tissue injury (3). This manifests as acute organ dysfunction and infection, potentially progressing to multiple organ failure, acidosis, and even death (2). Cardiac dysfunction was the most prevalent form of acute organ dysfunction. Acute organ dysfunctions most strongly associated with in-hospital mortality involved the neurological system (OR = 1.86; p < 0.001), respiratory system (OR = 1.43; p < 0.001) and heart (OR = 1.31; p < 0.001) (4). Part of patients surviving the acute phase, but then develop into persistent chronic critical illness characterized by prolonged inflammation, immunosuppression, and organ damage, maintaining sustained the risk of death (2).

Previous research typically divided the host response to sepsis into two distinct phases: pro-inflammation and immunosuppression (5). Alternative perspectives suggest these phases may coexist (6), though current investigations remain predominantly focused on the acute phase. During the acute phase, patients may develop hemolysis (7, 8), leading to systemic accumulation of hemoglobin (9, 10). Reactive oxygen/nitrogen species (ROS/RNS) can oxidize hemoglobin to methemoglobin (MtHb) (11). The inherent instability of circulating MtHb facilitates rapid heme release following its formation (11).

In circulation, heme that remains unbound or loosely associated with albumin or low-density lipoprotein (LDL) is called free heme, which exhibits cytotoxic properties (12). *Larsen R* et al. established homozygous HO-1 knockout mice (9). Compared with wild littermates, *Hmox1^−/−^* mice lack intracellular HO-1, with no disruption to heme binding to HPX, albumin or LDL. For this reason, heme in Hmox1*^−/−^* mice is defined as cell-free heme (9). Compared to mice subjected to low-grade cecal ligation and puncture (CLP, <20% mortality), those undergoing high-grade CLP (>90% mortality) demonstrate elevated plasma levels of free hemoglobin, higher concentrations of free heme, and reduced levels of heme-binding protein (HPX) (9). Administration of HPX to CLP mice significantly attenuates organ failure compared to IgG-injected controls (9). These findings indicate that free heme constitutes a critical pathogenic component in severe sepsis (9). Additionally, injection of cell-free heme into mice with low-grade CLP-induced sepsis led to progression to severe sepsis and aggravated histological organ damage (9). Likewise, renal damage was observed in wild-type mice receiving free heme (9), indicating high renal sensitivity to heme and reinforcing its role in tissue injury. Given that organ dysfunction has been formally incorporated into the Sepsis-3 definition (3), free heme may therefore be recognized as a key driver of sepsis deterioration and tissue damage.

Current research on the pathogenesis and progression of sepsis remains extensive, gradually filling in this field, yet specific therapeutic strategies targeting sepsis still remain lacking (13). Based on existing evidence linking heme to sepsis pathogenesis, it can be found that systematic investigations persist limitations and mechanistic questions regarding heme’s role in sepsis development are under solved, however, heme as a critical component in sepsis pathophysiology (9), retains significant potential as a targeted therapeutic candidate. To elucidate the association between free heme and sepsis progression, this review will systematically conclude mechanisms by which free heme exacerbates sepsis, focusing on its direct pathological effects and involvement in regulated cell death pathways, including apoptosis, pyroptosis, necroptosis, ferroptosis, and PANoptosis. Furthermore, we will delineate potential therapeutic targets within these mechanisms, aiming to facilitate the development of novel targeted therapies.

## 2 Heme catabolism

### 2.1 Overview of the heme and the heme oxygenase

In the context of sepsis, extravascular hemolysis occurs, where cell-free hemoglobin is oxidized to MtHb, which in turn rapidly releases free heme. Heme can enter the cytoplasm via multiple pathways, including heme-responsive gene 1 (HRG1) and the heme transporter heme carrier protein 1 (HCP1) (14). *In vivo*, heme catabolism is mediated by heme oxygenase (HO). Mammals possess two functional HO isoforms: HO-2 is constitutively expressed in specific tissues, whereas HO-1 is a stress-inducible isozyme (15).

*HMOX1* (the gene encoding heme oxygenase-1) binds to enhancer sequences of numerous transcription factors responsible for tissue redox homeostasis, including activator protein-1 (AP-1), nuclear factor-κB (NF-κB), hypoxia-inducible factors (HIFs) and nuclear factor erythroid 2-related factor 2 (NRF2) (16). Accordingly, all these transcription factors exert regulatory activity on HO-1 expression via their cognate enhancer elements. Among these, the transcription factor NRF2 is the master regulator of the oxidative stress response and is negatively regulated by BTB and CNC homology 1 (BACH1) (17). Recent studies have identified TANK-binding kinase 1 (TBK1) acts as an upstream regulator of BACH1 that promotes BACH1 degradation through both phosphorylation-dependent/-independent mechanisms, in a heme-independent manner (18).

### 2.2 Overview of the heme catabolism

Heme degradation proceeds via a multi-step mechanism (19, 20). The first step involves the binding of heme to ligand amino acid residues of HO-1, such as His-25 (20). In the second step, heme is oxidized to α-hydroxyheme, a process that requires oxygen and reducing equivalents supplied by CPR (21). The third step generates α-verdoheme, accompanied by the release of carbon monoxide. The fourth step produces biliverdin-iron chelate, which also requires oxygen and electrons from CPR (22). In the final step, the iron within the biliverdin-iron chelate is reduced by CPR, ultimately releasing ferrous ions and biliverdin (23). Numerous additional mechanisms underlying heme degradation have been reported, and here we mainly describe the pathways associated with Poly(rC)-binding protein 2(PCBP-2).

### 2.3 The pathways associated with PCBP2

Studies have demonstrated that PCBP-2, a member of the PCBP family, specifically binds to HO-1 primarily via its KH3 domain and competes with CPR for the same binding site (24). The C-terminus of PCBP-2 interacts with FPN1 while its N-terminus binds to DMT1, thereby mediating ferrous ion transport (24).

Fumio Kishi et al. proposed a hypothetical functional model illustrating how PCBP-2 participates in heme catabolism through HO-1. The initial step of heme degradation is the binding of heme to HO-1 (20). Heme occupies the heme-binding pocket of HO-1, with its iron atom directly interacting with ligand residues such as His-25, which triggers conformational changes in the enzyme. This structural alteration facilitates the association between CPR and HO-1, leading to the dissociation of PCBP-2 that previously occupied the same binding region. In the final step of heme degradation, CPR reduces iron within the biliverdin-iron chelate to generate ferrous ions and biliverdin (23). Afterwards, CPR dissociates from HO-1, and the iron-free PCBP-2 re-binds to HO-1 to shuttle the newly produced ferrous ions. Ultimately, PCBP-2 detaches from HO-1 along with ferrous ions and delivers these ions to downstream proteins including FPN1 and DMT1 (24).

## 3 Direct effects of heme

### 3.1 Cytotoxic effects

The amphiphilic nature of heme enables its preferential partitioning into nonpolar niches, including lipoproteins and lipid membranes (11). Consequently, heme can destabilize cellular membranes and increase its permeability (11). Upon embedding its porphyrin ring into lipid bilayers, heme increases cellular susceptibility to oxidants, exacerbating lipid peroxidation (11). When targeting erythrocytes, this process manifests as hemolysis, further promoting hemoglobin and heme release, thereby establishing a feedforward loop that generates excessive free heme (25).

The ferrous iron (Fe^2+^) at the porphyrin core participates in Fenton reactions to generate ROS/RNS (12). In the presence of ROS, hemoglobin is oxidized to MtHb, converting Fe^2+^ into ferric iron (Fe^3+^). Rapidly, MtHb releases heme, then degraded by heme oxygenase (HO) to liberate Fe^3+^ (11). Fe^3+^ reacts with hydrogen peroxide (H_2_O_2_) to produce hydroxyl radicals (•OH) (11). These radicals propagate lipid peroxidation, ultimately triggering cascading oxidative damage—a central mechanism underlying the direct cytotoxicity of heme (11).

### 3.2 Pro-inflammatory effects

Heme activates endothelial cells to induce adhesion molecules such as ICAM-1 and VCAM-1, facilitating neutrophil adhesion to vascular endothelium and subsequent transmigration (11, 25). Additionally, heme acts as a chemotactic agent or induces macrophage-derived chemokines like leukotriene B4 (LTB4) (26), further promoting neutrophil recruitment. Heme also stimulates macrophages to produce cytokines including TNF, KC (27), and IL-1β. Notably, TNF contributes to necroptosis activation (discussed later). Furthermore, heme oxidizes LDL and scavenges nitric oxide (NO), impairing vascular homeostasis (12, 25, 28).

### 3.3 Impairment of disease tolerance

Host immunity against pathogens primarily operates through resistance mechanisms—targeting pathogens for elimination or containment to mitigate their assault (29, 30). However, these processes often inflict collateral tissue damage. To balance this damage, a complementary defense strategy termed *disease tolerance* emerges, which limits host damage without altering pathogen burden (29, 30).

Studies demonstrate that intraperitoneal administration of heat-killed bacteria combined with heme induces 100% mortality in *Hmox1*^+/+^ mice, whereas heat-killed bacteria with DMSO results in only 12.5% mortality (9). This indicates that free heme impairs the disease tolerance capacity, exacerbating severe sepsis independently of pathogen load (9).

## 4 Heme-induced apoptosis

### 4.1 Overview of apoptosis

Apoptosis is a non-inflammatory programmed cell death primarily mediated by caspase activation (31, 32), characterized morphologically by cell shrinkage, chromatin condensation, nuclear fragmentation, and apoptotic body formation. Caspase-3/7 are recognized as the executioner proteases critical for apoptotic progression (32), with multiple upstream pathways converging on their activation (33).

**Figure 1:**
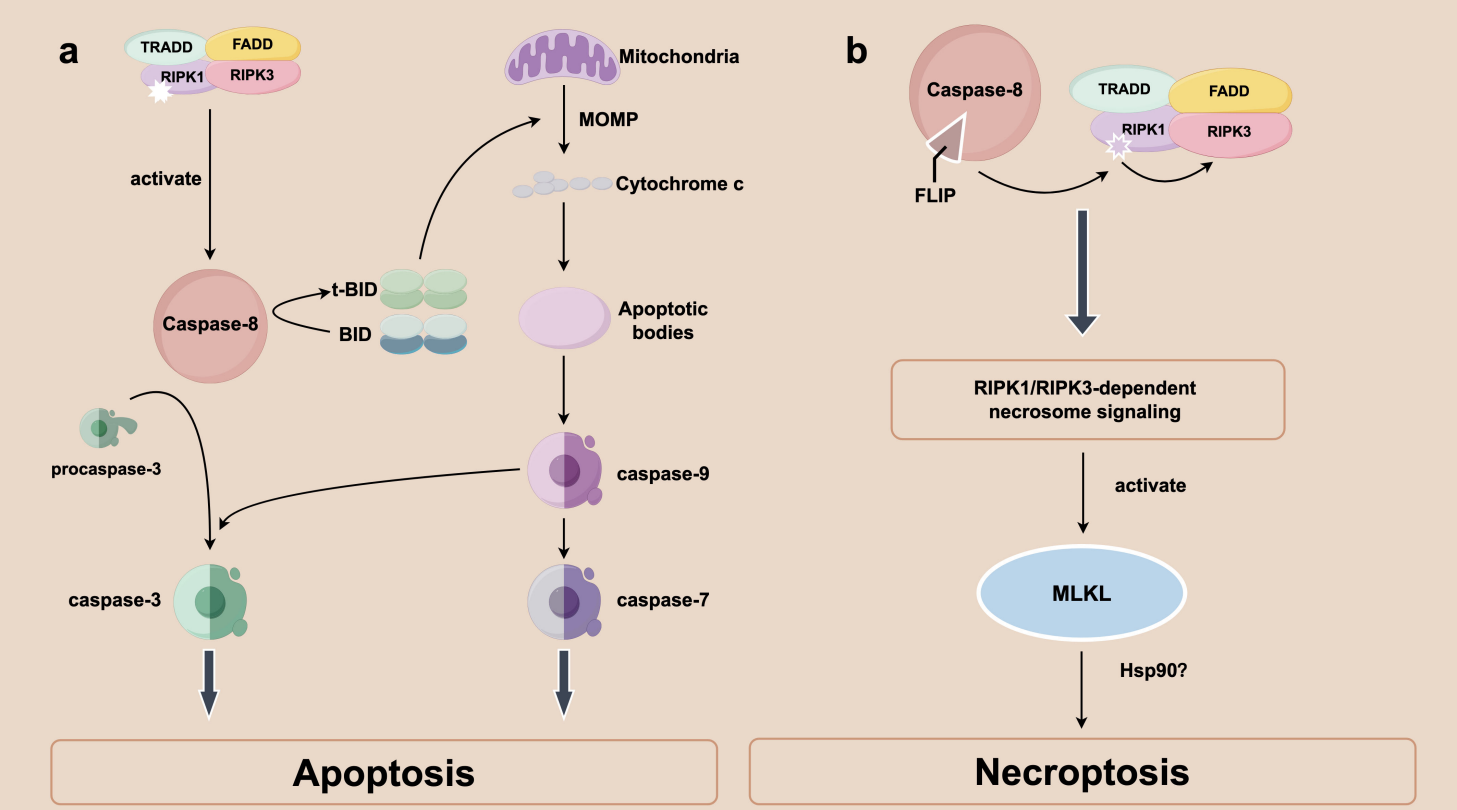
**(a)** Extrinsic apoptosis is initiated by the activation of cell surface death receptors or dependence receptors. Taking TNF binding to death receptor TNFR as an example, this leads to the recruitment of adaptor proteins such as TRADD and FADD, which subsequently activate caspase-8. Activated caspase-8 can directly cleave pro-caspase-3 to execute apoptosis. Alternatively, it cleaves the BH3-interacting domain death agonist (BID) into truncated BID (tBID). tBID then translocates to the mitochondrial membrane, promoting pore formation and the release of cytochrome c, thereby initiating the intrinsic apoptotic pathway. Intrinsic apoptosis begins with mitochondrial outer membrane permeabilization (MOMP). Damaged mitochondria release apoptotic factors, including cytochrome c, into the cytoplasm. This is followed by the formation of the apoptosome. Caspase-9 undergoes auto-activation within this complex and subsequently cleaves and activates pro-caspase-3 and -7, leading to apoptotic cell death. **(b)** Using TNF binding to TNFR1 as an example, ligand engagement induces conformational changes in TNFR1, leading to the formation of Complex I, which activates the NF-κB pathway. Subsequently, TNFR1 is internalized and assembles into Complex II, containing TRADD, FADD, caspase-8, RIPK1, and RIPK3. The NF-κB pathway induces the expression of FLIP, which then binds to caspase-8, forming an inactive heterodimer and thereby suppressing caspase-8 activity. Under conditions of caspase-8 inhibition, RIPK1 exposes its RHIM domain and interacts with RIPK3 to form an amyloid signaling complex, known as the necrosome. This complex serves as a platform for phosphorylating and activating MLKL. Activated MLKL, potentially facilitated by Hsp90 in its oligomerization and membrane translocation, ultimately executes necroptosis by disrupting plasma membrane integrity. Image by Figdraw.

Intrinsic apoptosis initiates with irreversible mitochondrial outer membrane permeabilization (MOMP) (34). Damaged mitochondria release apoptogenic factors such as cytochrome c from the intermembrane space, leading to apoptosome assembly. Caspase-9 undergoes autoactivation through homodimerization or hetero-/multimerization with Apaf-1, subsequently cleaving and activating pro-caspase-3/7 to execute apoptosis (35) (**Figure 1a**).

Extrinsic apoptosis is triggered by cell surface death receptor activation (34). For example, tumor necrosis factor (TNF) binding to TNFR recruits TRADD and FADD34, activating caspase-8 (36). Caspase-8 directly cleaves pro-caspase-3 to induce apoptosis or processes BID into truncated BID (tBID), which permeabilizes mitochondria to release cytochrome c, thereby engaging the intrinsic pathway (35) (**Figure 1a**).

Additional mechanisms, such as granzyme B-dependent caspase activation, may also activate caspase-3/7 to initiate apoptosis.

### 4.2 Synergistic induction of apoptosis by heme and co-signals

Isolated treatment with low-dose heme (5 μM) or subtoxic TNF (5 ng/mL) fails to induce cytotoxicity in hepatocytes. However, co-treatment triggers >25% cell death with apoptotic hallmarks, indicating that free heme can sensitize hepatocytes to TNF-mediated apoptosis (37). This cytotoxic effect correlates with caspase-3 cleavage (37, 38).

Further studies demonstrate that heme synergizes with other cytotoxic agonists, including Fas ligand, H_2_O_2_, and ONOO^-^, to promote programmed cell death (9). Notably, several of these agonists are implicated in severe sepsis pathogenesis (9). These findings suggest that heme cooperates with cytotoxic agonists to promote apoptosis, and this way may exacerbate sepsis progression.

## 5 Heme-induced inflammatory regulated cell death

### 5.1 Mechanisms of heme-induced necroptosis

#### 5.1.1 Overview of necroptosis

Necroptosis is a form of regulated cell death triggered by specific death receptors detecting perturbations in extracellular or intracellular microenvironments (34). In the canonical TNF-TNFR1 pathway, TNF binding induces conformational changes in TNFR1 to form Complex I, activating the NF-κB pathway (39). Subsequently, internalized TNFR1 assembles Complex II containing TRADD, FADD, caspase-8, RIPK1, and RIPK3 (39). NF-κB signaling induces FLIP, which binds caspase-8 as an inactive heterodimer (36) (**Figure 1b**). RIPK1 undergoes ubiquitination, deubiquitination, and phosphorylation, exposing its RHIM domain under caspase-8 inhibition to interact with RIPK3 (36). With HSP90 and CDC37 assistance, RIPK1/RIPK3 form a necrosome, phosphorylating MLKL to induce its homooligomerization and translocation to the plasma membrane. This increases membrane permeability, executing necroptosis (40). Current evidence suggests HSP90 facilitates MLKL oligomerization and conformational changes (41, 42), though the precise mechanism of MLKL-mediated membrane disruption remains incompletely understood (40) (**Figure 1b**).

Notably, RIPK1 is dispensable for necroptosis in some contexts (43), but remains essential in the well-characterized TNF-TNFR pathway.

#### 5.1.2 Heme and necroptosis

Heme activates TLR4 through a mechanism distinct from LPS, establishing that heme can influence cellular functions as an extracellular signaling molecule via receptor engagement (27). Structural studies reveal heme binds W23 and Y34 residues on MD-2 to initiate TLR4 signaling (44). Via TLR4-dependent pathway, TNF can be induced in macrophages, which binds TNFR to drive RIPK1/RIPK3-dependent macrophage necroptosis (45). Concurrently, heme generates ROS via Syk-, NADPH oxidase-, and mitochondrial electron transport chain-dependent mechanisms independent of TLR4 (11, 46, 47). Sustained JNK activation by ROS establishes a feedforward loop, amplifying oxidative stress and promoting cell death (45).

### 5.2 Mechanisms of heme-induced pyroptosis

#### 5.2.1 Overview of pyroptosis

Pyroptosis, a regulated cell death modality linked to innate immunity, is triggered by perturbations in cellular homeostasis (34). Distinct from apoptosis, pyroptosis features inflammatory caspase involvement, plasma membrane pore formation with cytoplasmic content release, DNA damage and so on (48).

The activation platform for inflammatory caspases is called inflammasome, a cytosolic multiprotein complex (49, 50). Three pathways are known to activate inflammasomes: the canonical NLRP3 inflammasome pathway, the non-canonical inflammasome pathway, and the alternative NLRP3 inflammasome pathway.

##### 5.2.1.1 Canonical NLRP3 inflammasome activation pathway

Canonical NLRP3 inflammasome activation requires two steps: priming and activation.

**Figure 2:**
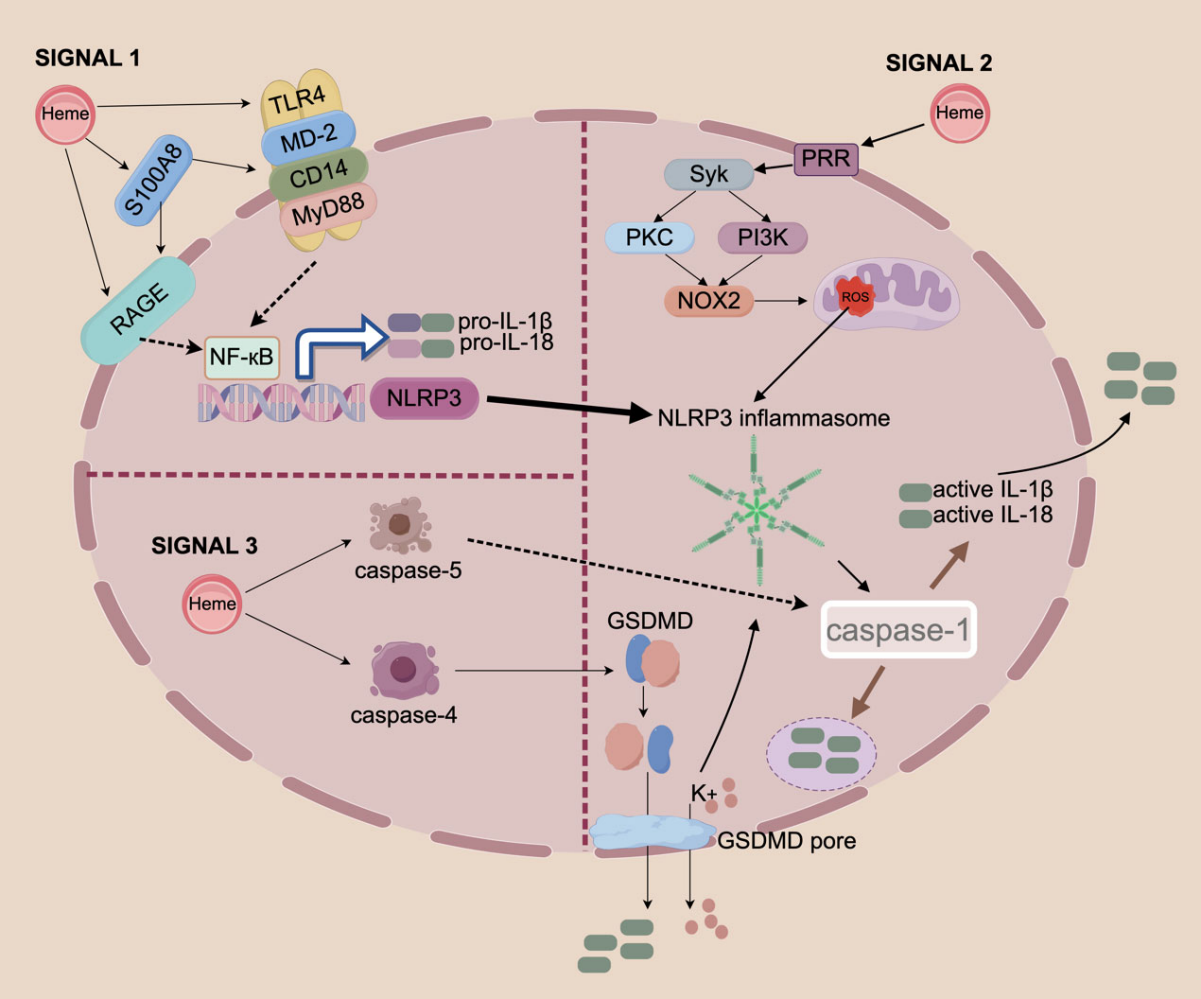
Step 1 initiates the activation of the canonical inflammasome pathway. Downstream products of heme serve as Signal 1, activating the NF-κB pathway via RAGE and MD-2, leading to the upregulation of NLRP3, caspase-1, and pro-IL-1β. Step 2 involves NLRP3 activation. Although a consensus model for NLRP3 activation is lacking, known triggers include multiple upstream signals (Signal 2), with heme-mediated activation involving K^+^ efflux and ROS production. NLRP3 oligomerizes and sequentially assembles with ASC and caspase-1 to form the NLRP3-ASC-caspase-1 complex, completing inflammasome assembly. This complex cleaves pro-caspase-1 into its active form, which then processes pro-inflammatory cytokines IL-1β and IL-18 into their mature forms. Simultaneously, active caspase-1 cleaves gasdermin D (GSDMD), generating the N-terminal fragment (GSDMD-N). GSDMD-N translocates to the plasma membrane, forming pores that facilitate the release of IL-1β, IL-18, and other cytoplasmic contents, thereby inducing pyroptosis and further promoting potassium efflux. Heme can also activate the non-canonical inflammasome pathway as Signal 3, and is currently the only known DAMP that stimulates this pathway. It triggers intracellular caspase-4 and caspase-5 activation, which cleave GSDMD to generate GSDMD-N. However, their functions are distinct: caspase-4 primarily mediates GSDMD cleavage, whereas caspase-5 predominantly regulates caspase-1 activation. Together, they coordinate the release of mature IL-1β. Image by Figdraw.

The first step is the priming of inflammasome. TNF-α (as Signal 1) activate the NF-κB pathway (41) (**Figure 2**), upregulating the expression of NLRP3, caspase-1, and pro-IL-1β (51). NF-κB signaling also induces NLRP3 post-translational modifications (PTMs)—including ubiquitination, phosphorylation, and SUMOylation—stabilizing NLRP3 in an autoinhibited but signal-sensitive state (52).

The second step is the activation of NLRP3. NLRP3 is activated by microbial infections mediated by pathogen-associated molecular patterns (PAMPs), sterile inflammation mediated by damage-associated molecular patterns (DAMPs) or environmental stimuli. Although there is no consensus model exists, known activators include upstream signals (Signal 2) such as K^+^ efflux, lysosomal rupture, mitochondrial dysfunction, metabolic alterations, and trans-Golgi disassembly (52, 53).

Upon DAMP/PAMP sensing, NLRP3 oligomerizes via its NACHT domain, recruiting ASC to form ASC specks (54). ASC subsequently recruits pro-caspase-1 (via CARD-CARD interactions) to assemble the NLRP3-ASC-caspase-1 complex, completing NLRP3 inflammasome formation. The inflammasome induces pro-caspase-1 autoprocessing into active caspase-1, which cleaves pro-IL-1β and pro-IL-18 into their mature forms (55). Concurrently, caspase-1 cleaves gasdermin D (GSDMD) to generate GSDMD-N-terminal fragments (GSDMD-N) (ref.44). Oligomerized GSDMD-N forms plasma membrane pores, releasing IL-1β, IL-18, and other cytoplasmic contents—a hallmark of pyroptosis (55). This process further amplifies K^+^ efflux, establishing a feedforward loop (**Figure 2**).

##### 5.2.1.2 Non-canonical inflammasome activation

Activation of caspase-4, caspase-5 (human isoforms), and caspase-11 (murine ortholog) constitutes non-canonical inflammasome activation. Caspase-11 serves as the principal effector in LPS-induced lethal sepsis (56, 57). Extracellular LPS activates TLR4, upregulating caspase-11 expression via type I interferon responses and the Cpb1-C3-C3aR axis (58). Cytosolic LPS binding triggers caspase-11 oligomerization and autoproteolytic activation (59). Activated caspase-11 opens pannexin-1 channels to induce K^+^ efflux, driving NLRP3 inflammasome activation and subsequent IL-1β/IL-18 maturation (55). GSDMD is the key pyroptotic substrate of caspase-11 *in vivo* (57), with caspase-11-mediated cleavage of GSDMD generating pore-forming N-terminal fragments (GSDMD-N) (57, 60, 61). With these plasma membrane pores, IL-1β/IL-18 can be released and facilitate pyroptosis.

##### 5.2.1.3 Alternative NLRP3 inflammasome pathway

The alternative NLRP3 inflammasome pathway bypasses pyroptotic body formation and GSDMD-mediated membrane pore formation (55, 62). Thereby avoiding cytokine release via pyroptosis. As the pathway does not directly induce pyroptosis, we will not detail this here.

#### 5.2.2 Heme and pyroptosis

##### 5.2.2.1 Heme and the canonical NLRP3 inflammasome pathway

Heme-induced downstream products may serve as Signal 1 to prime inflammasome activation (**Figure 2**). TLR4 is required for heme-mediated NF-κB activation and subsequent TNF-α release (53), with coreceptors CD14, MyD88, and MD-2 essential for TLR4 signaling (27, 44, 63). While heme binds MD-2 to initiate TLR4-driven inflammasome priming (44), no evidence confirms TLR4 activation by heme-MD-2 interaction alone (53). RAGE oligomerization requires heme binding to its V domain, activating the NF-κB pathway to upregulate IL-1β expression. Given RAGE’s predominant expression in the lungs, heme-RAGE interactions may critically drive pulmonary inflammation (64); however, whether RAGE activation triggers heme-induced inflammasome pathways remains unclear (53). Additionally, heme enhances expression of the cofactor S100A8 protein, which acts as a DAMP to promote IL-1β production via TLR4 and RAGE (65).

Heme generates weak priming signals for inflammasome activation (65–67), but can synergize with PAMPs such as LPS (67), amplifying IL-1β mRNA expression under septic conditions involving concurrent hemolysis and infection.

Heme-induced NLRP3 inflammasome activation involves K^+^ efflux and ROS production (Signal 2) but excludes lysosomal rupture, cathepsin release, heme internalization, ATP release, or P2X7 activation (68) (**Figure 2**).

Heme triggers mitochondrial ROS (mtROS) generation in macrophages, dependent on spleen tyrosine kinase (Syk) (68). Syk likely mediates mtROS formation via downstream protein kinase C (PKC) and PI3K signaling (69). PKC and PI3K pathways activate NADPH oxidase (NOX), which is required for heme-induced IL-1β release (70). Notably, NOX2-derived mtROS are uniquely required for inflammasome activation, as NOX deficiency abolishes mtROS generation and subsequent caspase-1/IL-1β processing (68). Other NLRP3 activators (e.g., ATP) also require mtROS for IL-1β cleavage but act independently of Syk and NOX2 (68).

##### 5.2.2.2 Heme and the non-canonical inflammasome pathway

Heme uniquely activates the non-canonical inflammasome pathway (Signal 3) as the only known DAMP stimulating this cascade (**Figure 2**). In human macrophages, heme activates caspase-1, caspase-4, and caspase-5 (66), though direct binding to caspase-4/-5 remains unconfirmed (53). Heme triggers caspase-4/-5-dependent GSDMD cleavage into GSDMD-N. Functional divergence exists: caspase-4 primarily regulates GSDMD processing, while caspase-5 modulates caspase-1, jointly coordinating IL-1β maturation and release (66).

### 5.3 Mechanisms of heme-induced ferroptosis

#### 5.3.1 Overview of ferroptosis

Ferroptosis is a regulated cell death triggered by specific perturbations in the intracellular microenvironment, dependent on ROS and iron (34), and independent of apoptotic or necroptotic machinery (71). Fe^3+^ enters endosomes via the transferrin/transferrin receptor (Tf/TfR) pathway, where it is reduced to Fe^2+^ by STEAP2 metalloreductase and transported into the cytosol through divalent metal transporter 1 (DMT1), forming the labile iron pool (LIP) (72, 73). As the LIP expands, Fe^2+^ undergoes Fenton reactions to generate ROS, driving lipid peroxidation and ultimately ferroptosis (73, 74). Ferritin-bound iron, degraded via nuclear receptor coactivator 4 (NCOA4)-mediated ferritinophagy (75, 76), further elevates the LIP, exacerbating ferroptosis.

The Xc^-^ system and glutathione peroxidase 4 (GPX4) critically regulate ferroptosis. The Xc^-^ system imports cystine for glutathione (GSH) synthesis, while GPX4 catalyzes GSH oxidation to glutathione disulfide (GSSG), suppressing lipid peroxidation (73). Agents such as erastin, RSL3, DPI7, FIN56, and FINO2 induce ferroptosis by targeting Xc^-^ and GPX4 pathways (77).

#### 5.3.2 Heme and ferroptosis

Post-hemolysis, extracellular hemoglobin is oxidized to release heme, which binds haptoglobin (HPX) and enters cells via the CD91 receptor (38). Intracellular HO-1 degrades heme, generating Fe^2+^—a major source of intracellular labile iron, then induce oxidative stress in cells.

Ferritin heavy chain (Fth) exerts ferroxidase activity, oxidizing Fe^2+^ to Fe^3+^ (78), thereby mitigating oxidative stress. Fth enhances host survival without altering pathogen burden, indicating its role in disease tolerance (78). Additionally, Fth promotes gluconeogenesis to elevate blood glucose, counteracting heme-induced hypoglycemia to improve survival (78). However, during severe hemolysis, iron overload surpasses the compensatory capacity of Fth, accelerating ferroptosis. In the presence of TNF, ROS production sustains JNK activation, with JNK1 promoting ferritin degradation to reduce Fth levels and expand the LIP (79).

Iron participates in critical metabolic processes of pathogens during sepsis, potentially facilitating bacterial proliferation and enhancing virulence (80). Host defense mechanisms restrict extracellular iron availability by sequestering serum iron intracellularly, including hepcidin (HAMP)-mediated degradation of ferroportin-1 (FPN), IFN-γ-induced suppression of FPN transcription, and Tf/TfR-mediated iron uptake (72, 73, 81).

While hypoferremia is crucial for limiting iron availability and immune-driven resistance to extracellular pathogens, sepsis pathophysiology involves cytokine surges and TLR4-LPS signaling that upregulate hepcidin, degrading FPN—the sole known iron exporter—and inducing intracellular iron overload (81). This drives excessive ROS production and lipid peroxidation (82), culminating in large ferroptosis.

Sepsis-3 defines sepsis as life-threatening organ dysfunction (9), with studies correlating tissue injury and multi-organ dysfunction syndrome (MODS) severity with iron accumulation (83, 84). Ferroptosis inhibitors like UAMC-3203 demonstrate robust organ-protective effects (80).

### 5.4 Mechanisms of heme-induced PANoptosis

#### 5.4.1 Overview of PANoptosis

The co-regulation and molecular crosstalk among apoptosis, pyroptosis, and necroptosis led to the conceptualization of “PANoptosis” in 2019 (74), classified as a form of programmed cell death (85, 86).

PANoptosis occurs broadly during bacterial, fungal, and viral infections (87). It integrates features of pyroptosis, apoptosis, and necroptosis but cannot be fully explained by any of these pathways individually, representing a unique lytic, innate immune-inflammatory cell death pathway (88).

For instance, caspase-8 not only serves as a molecular switch for apoptosis and necroptosis but also mediates GSDMD cleavage to induce pyroptosis under TAK1 inhibition (87), highlighting cross-regulatory mechanisms among these pathways. In a rat model of sepsis-associated encephalopathy (SAE), necroptosis activation suppresses apoptosis and pyroptosis, whereas necroptosis inhibition triggers compensatory activation of apoptosis and pyroptosis (89), demonstrating reciprocal compensatory mechanisms in PANoptosis (87). Furthermore, cytokine release by host cells post-pathogen challenge forms a positive feedback loop with PANoptosis, amplifying its progression (87).

To date, five PANoptosome complexes—ZBP1, AIM2, RIPK1, NLRP12, and the recently identified NLRC5—have been shown to induce PANoptosis. Emerging evidence suggests that NLRP12- and NLRC5-mediated PANoptosomes play critical roles in pathological conditions involving free heme release (88, 90).

#### 5.4.2 Heme and PANoptosis

##### 5.4.2.1 NLRP12 PANoptosome

**Figure 3:**
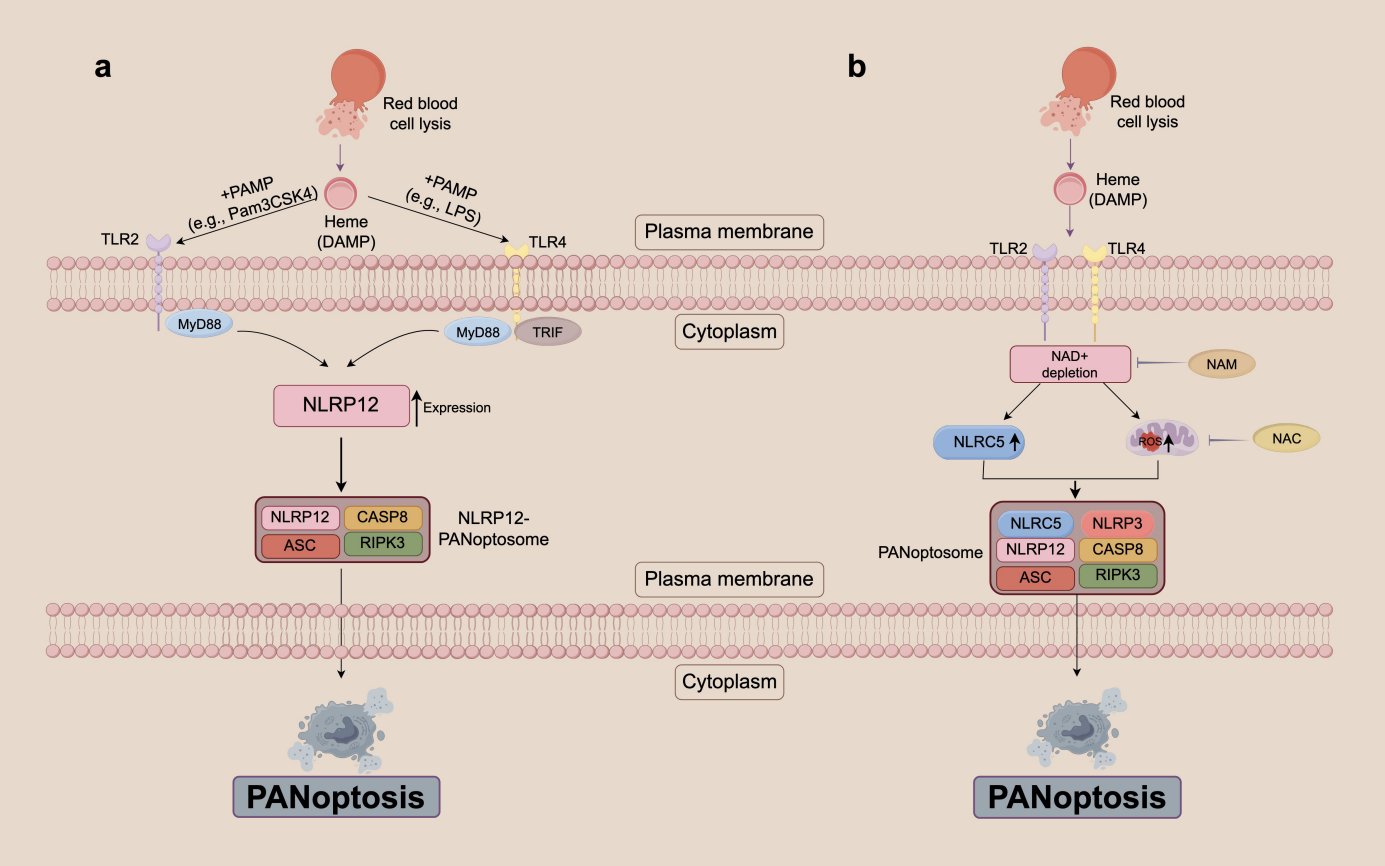
**(a)** The combined stimulation of heme with PAMPs or TNF activates TLR2/4 and MyD88 signaling, leading to upregulated NLRP12 expression. NLRP12 then assembles with caspase-8 into the NLRP12 PANoptosome, a key driver of inflammasome formation and inflammatory cell death. **(b)** NLRC5—whose expression is modulated by TLR signals and NAD^+^ levels—responds to heme and PAMPs, interacts with NLRP12, and forms the NLRC5 PANoptosome, ultimately inducing PANoptosis. Image by Figdraw.

Under heme+Pam3 stimulation, *Nlrp12*^−/−^ cells exhibited reduced cell death compared to *Nlrp3*^−/−^ cells, indicating NLRP12 specifically senses heme to drive a distinct death pathway beyond pyroptosis. Mechanistically, NLRP12 responds to heme combined with PAMPs or heme combined with TNF via TLR2/4 and MyD88, orchestrating inflammasome assembly and inflammatory cell death through caspase-8 (90) (**Figure 3a**). In hemolysis models, *Nlrp12*^−/−^mice showed significant protection, with attenuated acute kidney injury and mortality compared to wild-type controls. These mechanisms operate similarly in infections and inflammatory diseases associated with hemolysis (90).

##### 5.4.2.2 NLRC5 PANoptosome

Emerging studies identify NLRC5, NLRP12, and ASC as components of a PANoptosome complex activated by heme+PAMP stimulation. In malaria patients, NLRC5 expression is markedly upregulated in whole blood, CD71+ cells, and sickle cell disease (SCD) monocytes, correlating with elevated circulating free heme levels in hemolytic disorders. Beyond TLR2/4 signaling, NAD^+^ levels also regulate NLRC5 expression (88).

Specifically, NLRC5 interacts with NLRP12 to form the NLRC5 PANoptosome in response to heme+PAMPs, with NLRC5 expression modulated by both TLR signaling and NAD+ homeostasis (88) (**Figure 3b**).

Current research on heme-induced PANoptosis remains limited, primarily focusing on innate immune cells and macrophages. Whether NLRP12/NLRC5 PANoptosome-driven PANoptosis induced by free heme occurs in non-immune cells remains unknown. Nevertheless, NLRP12 and NLRC5 represent promising therapeutic targets for hemolysis-associated infections and inflammatory diseases.

##### 5.4.2.3 Alternative mechanism of PANoptosis activation

Distinct from the mechanism in which heme triggers PANoptosome formation by activating NLRP12 and NLRC5, Li, T. et al. proposes a novel regulatory pathway (91). Combined exposure to heme and bacterial infection facilitates the translocation of cleaved GSDMD (cGSDMD) to mitochondria via PLC-γ, ultimately inducing mitochondrial damage (91). Mitochondrial injury not only initiates GSDMD-dependent pyroptosis, but also activates RIPK-MLKL-mediated necroptosis and caspase-dependent apoptosis (91). These interconnected events collectively drive a synergistic cell death pattern defined as PANoptosis (91). The precise molecular mechanism by which PLC-γ regulates the mitochondrial translocation of cGSDMD remains poorly understood and warrants further investigation.

## 6 Therapeutic targets

### 6.1 Targeting heme production and catabolic pathways

#### 6.1.1 Exogenous haptoglobin supplementation

Septic patients with hemolysis often receive packed red blood cell (PRBC) transfusions for supportive care. However, this intervention may exacerbate hemolysis (as previously described). Hemoglobin released from lysed erythrocytes partially binds to plasma haptoglobin (Hp), forming Hb: Hp complexes that undergo CD163 receptor-mediated endocytosis and intracellular degradation (38). Unbound hemoglobin exceeding the binding capacity of Hp rapidly oxidizes to release free heme, inducing cellular and tissue damage.

Augmenting circulating Hp levels may mitigate free heme toxicity to host. In guinea pig and canine sepsis models, Hp administration attenuates the detrimental effects of Hb and its degradation products on renal and vascular systems, reduces LPS-induced cardiomyocyte toxicity, and improves survival (92–94). Unresolved challenges include interspecies differences between preclinical models and human pathophysiology, as well as bacterial pathogens exploiting Hb: Hp complexes for iron acquisition, potentially compromising exogenous Hp efficacy (95). Despite its promise, Hp-based therapy requires further optimization and validation.

#### 6.1.2 Enhancing free heme intracellular uptake

Heme dissociates from hemoglobin and transiently associates with albumin, HDL, LDL, or α1-microglobulin before binding hemopexin (Hx) for CD91 receptor-mediated cellular internalization (38), followed by HO-mediated degradation.

Exogenous Hx reduces endothelial heme burden in murine models (96), and decreases circulating free heme levels (94). Experimental data show Hx-treated animals exhibit elevated aortic endothelial NOS mRNA expression and enzymatic activity (96), indicating enhanced NO bioavailability. NO also exerts anti-inflammatory, anti-proliferative, and disease tolerance-promoting effects (97), collectively reducing tissue inflammation and improving survival.

#### 6.1.3 Upregulating HO-1 expression

Nuclear factor erythroid 2-related factor 2 (NRF2) serves as the primary upstream regulator of *Hmox1*. *In vitro* studies identify natural compounds—such as curcumin, caffeic acid phenethyl ester, resveratrol, and quercetin—that enhance HO-1 expression via NRF2 activation (98). Pharmacological agents like dimethyl fumarate (DMF) also upregulate HO-1 (98). However, elevated HO-1 activity increases the production of heme metabolites, and excessive ferrous ions released during this process may aggravate ferroptosis. Therefore, the threshold between moderate HO-1 induction and its overexpression remains to be further explored. Besides, their *in vivo* selectivity, efficacy, safety profiles, optimal dosing regimens, and delivery methods require systematic validation.

#### 6.1.4 Increasing of active heme metabolites

Therapeutic administration of active products derived from HO-1 has shown potential to alleviate inflammation-mediated organ injury (99).

Gaseous CO exerts prominent protective effects in sepsis models (97, 100), and confers beneficial impacts on multiple organs (101). CO is capable of preventing blood-brain barrier (BBB) disruption (10), which includes suppressing pro-inflammatory responses and pyroptosis (97). A phase I clinical trial of low-dose inhaled carbon monoxide (iCO) demonstrated that iCO treatment may exert favorable clinical outcomes in patients with sepsis-induced ARDS by reducing circulating mtDNA levels and preserving mitochondrial function, leading to improvements in respiratory parameters and secondary systemic endpoints (102). To date, most studies on CO focus on determining its safe dosage range (99). Large-scale clinical trials are still required to verify its therapeutic efficacy.

Current studies have revealed that biliverdin exerts protective effects against sepsis via regulating inflammatory mediators (103). It also elevates the 24-hour survival rate by alleviating pulmonary cell injury induced by endotoxemia (104). Nevertheless, no relevant clinical trials concerning biliverdin have been conducted so far, and its protective role in septic patients remains to be further clarified.

### 6.2 Targeting cell death pathways

#### 6.2.1 RIPK3

RIPK3, a hallmark mediator of necroptosis, forms a complex with RIPK1 upon activation, subsequently phosphorylating MLKL to increase plasma membrane permeability (36, 40). *Ripk3* deficiency exerts protective effects in murine CLP sepsis models, demonstrating RIPK3’s critical role in sepsis-mediated mortality (105). Although plasma RIPK3 levels do not exclusively correlate with necroptosis, elevated RIPK3 in ICU patients associates with higher in-hospital mortality and organ failure, suggesting necroptosis contributes to critical illness progression (106). This further implies circulating RIPK3 may serve as a biomarker for cellular injury and prognostic stratification in sepsis.

Necrostatin-1 (Nec-1), a RIPK1-specific inhibitor, mimics *Ripk3* knockout by attenuating intestinal morphological and functional damage, also improving outcomes in CLP models (105, 107). Safe pharmacological augmentation of Nec-1 activity could phenocopy *Ripk3* deficiency, suppressing pathological necroptosis.

#### 6.2.2 GSH/GPX4 axis

The Xc^-^ system and GPX4 regulate ferroptosis: Xc^-^ imports cystine for GSH synthesis, while GPX4 catalyzes GSH oxidation to glutathione disulfide (GSSG), inhibiting lipid peroxidation (73). Enhancing GSH/GPX4 axis activity thus mitigates ferroptosis by reducing lipid ROS accumulation.

Ferroptosis inhibitors—including ferrostatin-1, liproxstatin-1, and N-acetylcysteine (NAC)—elevate GSH/GPX4 levels, boost GPX4 enzymatic activity, and suppress lipid ROS generation (73). Additionally, iron chelators like deferoxamine (DFO) and deferiprone (DFP) can chelate iron ions, reduce circulating and LIP levels (73). Deferiprone, already approved for iron overload in thalassemia major, demonstrates clinical safety and could be repurposed—with optimized dosing—to attenuate post-hemolytic ferroptosis.

## 7 Discussion

Sepsis, a syndrome characterized by high incidence and mortality rates, remains a significant global burden despite recent data indicating declining prevalence (1–3), particularly in developing and underdeveloped regions (1). A hallmark of sepsis is infection, which elevates circulating free heme levels (7). This association has prompted investigations into the mechanistic role of free heme in sepsis progression. Through literature review, we summarized key mechanisms by which free heme exacerbates sepsis, including its direct effects (cytotoxicity, proinflammatory activity, and impairment of disease tolerance) and indirect effects mediated through regulated cell death pathways—apoptosis, pyroptosis, necroptosis, ferroptosis, and PANoptosis.

Free heme, as a critical damage-associated molecular pattern (DAMP), exerts a central driving role in multiple forms of regulated cell death through inducing cellular metabolic reprogramming (108, 109). It directly inhibits mitochondrial oxidative phosphorylation leading to cellular energy metabolism collapse, which activates RIPK1/RIPK3-mediated necroptosis (9, 45); triggers mitochondrial reactive oxygen species (ROS) burst to promote NLRP3 inflammasome assembly and GSDMD-mediated pyroptosis (9, 91); generates free ferrous ions via HO-1-mediated degradation, which initiate oxidative stress through the Fenton reaction while simultaneously inhibiting glutathione synthesis, ultimately triggering ferroptosis (110, 111). Furthermore, heme acts synergistically with bacterial infection to induce mitochondrial damage, and subsequent mitochondrial metabolic collapse triggers PANoptosome assembly, leading to PANoptosis (91). Aberrant activation of these heme-induced metabolism-cell death axes constitutes the key pathological basis for sepsis-induced organ injury and represents a major cause of mortality in sepsis patients. Subsequently, we sought to identify potential therapeutic targets based on the aforementioned mechanisms. However, the feasibility of these therapeutic targets and agents requires rigorous clinical validation, as current evidence predominantly derives from preclinical studies. Future research should prioritize clinical trials to evaluate efficacy, safety, and context-specific applicability.

## 8 Conclusions and perspectives

Hemolysis, initially characterized as a hallmark of malaria, has been increasingly recognized for its critical role in sepsis pathogenesis through the release of free heme. In this review, we delineate mechanisms by which free heme exacerbates sepsis, encompassing direct cytotoxic effects and the induction of regulated cell death pathways, including emerging modalities such as ferroptosis and PANoptosis. Furthermore, we propose potential therapeutic targets and highlight pharmacological agents with translational promise.

While updated clinical guidelines have reduced acute sepsis mortality, chronic sepsis—marked by persistent inflammation, immunosuppression, and cumulative organ damage—now accounts for a growing proportion of sepsis-related fatalities. Current research on heme-sepsis interactions predominantly focuses on acute-phase pathology, utilizing experimental models (e.g., LPS challenge and CLP) that simulate acute sepsis. Nevertheless, septic patients in clinical settings often have varied infectious origins and numerous comorbidities. Current mainstream disease models cannot perfectly mimic the actual clinical conditions, posing greater challenges to the research of sepsis therapies. Future investigations should integrate multiple disease models to prioritize elucidating heme’s role in chronic sepsis to refine pathophysiological understanding and develop stage-specific therapeutic strategies.
